# 

**Published:** 1894-04

**Authors:** 


### CONTENTS:

Article I.—To What Extent and Under What Conditions is the Collar-Crown a Cause of Pericemental Inflammation. By Prof. C. L. Goddard, A. M., D. D. S. - 529

" II.—Incidents Connected with the Trial of Prof. Webster. By Dr. Lester Noble. - - - - - - 532

" III.—Treating Infected Root-Canals with Kalium and Natrium. By Dr. Emil Schreier. - - - 536

" IV.—What are the Best Methods for Obtunding Sensibility of the Dentine by either Local or General Means? Should Arsenic Ever be Used? By Wm. H. Trueman, D. D. S. - - - - - - - - 542

" V.—Etiology of Pyorrhœa Alveolaris. By C. N. Peirce, D. D. S. - - - - - - - - - 552

" VI.—Anæsthetics, and the Physiology of the Heart. - 563

" VII.—A Few Things to be Remembered. By L. P. Haskell. 565

### EDITORIAL, Etc.

A Bill—Entitled an Act to Repeal and Re-enact with Amendments Article Thirty-two, of the Code of Public General Laws, Entitled "Dentistry." - - - - - - - - - - 567

The Baltimore College of Dental Surgery. - - - - 570

University of Maryland Dental Department. - - - - 571

Prof W. H. Eames, D. D. S. - - - - - - - - 572

Chicago Dental Society. - - - - - - - - 573

Illinois State Dental Society. - - - - - - - 573

### MONTHLY SUMMARY.

Repairing Vulcanite Plates. - - - - - - - 573

Lubricate Your Disks and Strips. - - - - - - 574

An Interesting Case. - - - - - - - - - 575

Paralysis Cured by Lancing the Gums, on a Little Girl of Twenty Months. - - - - - - - - - 576

Fitting Bands to Roots. - - - - - - - - - 576

